# Resection of Thymic Neuroendocrine Carcinoma Guided by Three-Dimensional Reconstruction: A Case Report

**DOI:** 10.3389/fmed.2022.859335

**Published:** 2022-07-06

**Authors:** Fang Liu, Hengxiao Lu, Liqian Chen, Junfeng Geng, Tongzhen Xu

**Affiliations:** ^1^Department of Respiratory Medicine, Weifang People’s Hospital, The First Affiliated Hospital of Weifang Medical University, Weifang, China; ^2^Department of Thoracic Surgery, Weifang People’s Hospital, The First Affiliated Hospital of Weifang Medical University, Weifang, China; ^3^Department of Pathology, Weifang People’s Hospital, The First Affiliated Hospital of Weifang Medical University, Weifang, China; ^4^Department of Thoracic Surgery, Shanghai Chest Hospital, Shanghai Jiao Tong University, Shanghai, China

**Keywords:** thymic small cell neuroendocrine carcinoma, three-dimensional reconstruction, surgical planning, uniport video-assisted thoracoscopy, prognosis, video-assisted thoracoscopy

## Abstract

Primary thymic small cell neuroendocrine carcinoma (SCNEC), which possesses a more aggressive biological behaviour, including invasion of proximal structures, local recurrence, and distant metastasis, is extremely rare. According to a previous literature report, only a few patients with this disease have been reported, compared to patients with distant metastasis of bones, lungs, spleen, liver, and adrenal glands (1, 2). The report data suggest that SCNEC is a highly malignant tumour compared to most other tumours of the human body. In this study, we presented the case of a patient who underwent surgery guided by three-dimensional reconstruction modelling before the operation. We were fully prepared for the resection of this tumour using three-dimensional reconstruction modelling, even after reading the computed tomography (CT) images that showed a closed relationship with the pericardium, the vein of the right middle lung lobe, and the phrenic nerve. All these features demonstrate that SCNEC is highly malignant. To date, there are no procedural reports for three-dimensional reconstruction modelling in malignant thymus tumours.

## Introduction

Thymic neuroendocrine carcinoma (NEC) is a malignant tumour originating from thymic tissue with neuroendocrine cells. It is rare in the clinic and accounts for 2–4% of mediastinal tumours (3). There were no specific symptoms or signs except for the invasion of surrounding tissues. The computed tomography (CT) displayed a large soft tissue mass with uniform density and local necrosis located in the anterior mediastinum. The enhanced CT scan also showed mild to moderate tumour enhancement. Thymic NEC possesses a more aggressive biological behaviour, including invasion of proximal structures, local recurrence, and distant haematogenous metastasis. Distant metastasis is often observed in the bones, lungs, spleen, liver, and adrenal glands. However, a study reported a patient with pancreatic metastasis resulting from thymic NEC.

## Case Report

**FIGURE 1:**
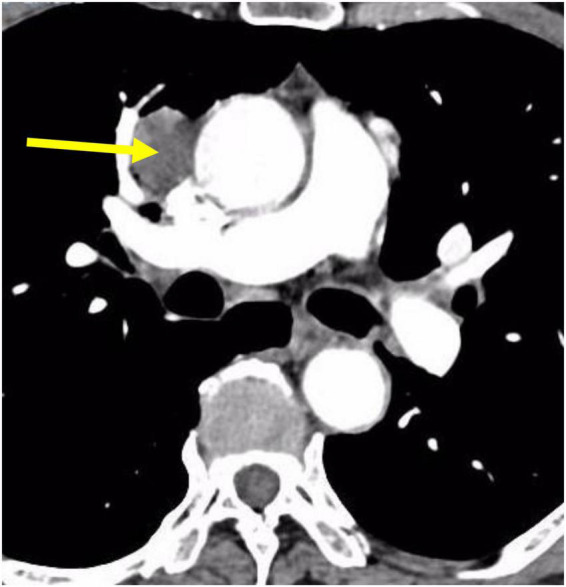
CT scans of the patient showing thymic neuroendocrine carcinoma with a closed relationship of proximal structures.

**FIGURE 2:**
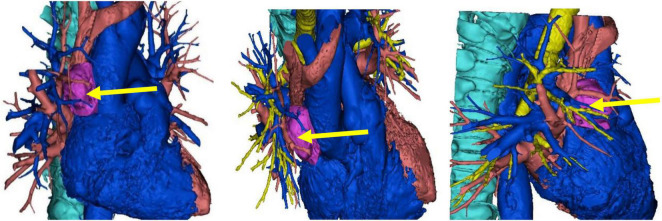
The three-dimensional reconstruction of this mass showing the relative positional relationship between the mass and the surrounding tissues.

**FIGURE 3:**
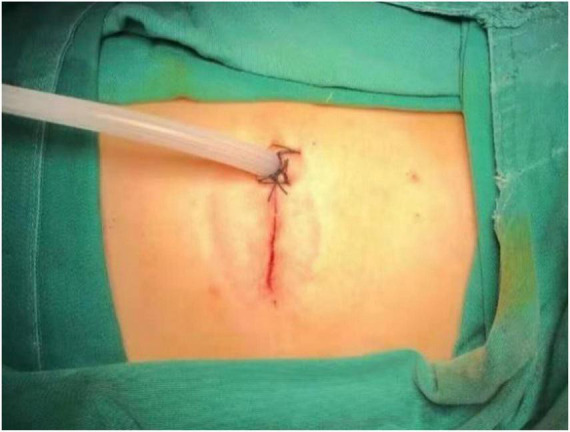
The patient underwent surgery through uniport video-assisted thoracoscopy (VATS).

A 71-year-old male patient was admitted to Weifang Peoples Hospital (The First Affiliated Hospital of Weifang Medical University, Weifang, China) in April 2021 with a 3.5 × 2.7 cm anterior mediastinal mass, which was observed on routine chest-enhanced CT. The patient had no symptoms, signs, or discomforts. The medical history of the patient revealed no special case earlier and no basic illness, including hypertension, diabetes, or coronary heart disease. A general physical examination was unremarkable. We completed various tests and inspections after hospitalisation. The enhanced CT scan showed that the soft tissue mass enclosed proximal structures, including lung tissue, pulmonary vein, pericardium, and phrenic nerve (Figure 1). We could tell that the operation would be very difficult and high risk. As a result, we performed three-dimensional reconstruction modelling of this mass as well as proximal structures to develop a safe surgical plan. Literature reports on three-dimensional reconstruction modelling applications in this area are rare. We could clearly see the relative positional relationship between the mass and the surrounding tissues, which provided us with a clear understanding of this mass. The mass clearly invaded the pericardium and right middle lobe (RML) tissue, with an extremely closed relationship between the pulmonary vein as well as the phrenic nerve (Figure 2). The patient underwent surgery to resect the mass through UniProt video-assisted thoracoscopy (VATS) (Figure 3). We performed a left lateral decubitus position surgery similar to the right lung lobectomy after communicating with Professor Junfeng Geng in the Department of Thoracic Surgery, Shanghai Chest Hospital, which is the most authoritative hospital on the chest in China. During the surgery, the tumour was observed to be located in the anterior mediastinum with an invasion of the adjacent structures, including the pericardium, RML and vein, superior vena cava, and phrenic nerve. The tumour invaded the pericardium and phrenic nerve, which did not remain intact. As a result, we resected part of the pericardium, RML, and phrenic nerve. We also performed sidewall moulding on the pulmonary vein, which was the main return path of the RML, instead of cutting it off. Eventually, the tumour was completely resected using three-dimensional reconstruction modelling preoperative planning that ensured the operation went smoothly. The postoperative pathological results were small cell neuroendocrine carcinoma (SCNEC) with a volume of 3.5 cm × 2.7 cm × 1.2 cm, accompanied by massive haemorrhage and local necrosis. Tumour thrombus was seen in the vessel and invaded the nerve. The staining results of immunohistochemistry were as follows: CK (+), CD117 (+), CD56 (+), Syn (+), CgA (+), CK7 (–), CK19 (–), CK20 (–), CK5/6 (–), P40 (–), TTF-1 (–), CD3 (–), CD20 (–), TdT (–), CD5 (–), and Ki-67 (30%).

## Discussion

Thymic neuroendocrine tumours are rare, with a variety of variations in their form, and are easily confused with other tumours. In 2004, the WHO carried out a new classification of thymic neuroendocrine tumours and put forward diagnostic criteria, which are of great significance to clinicopathological diagnosis (4). We first learn more about this rare disease from the following aspects:

### Definition

Thymic neuroendocrine tumours are epithelial tumours consisting mainly or entirely of neuroendocrine cells. It must be identified with thymus cancers and non-neuroepithelial tumours, especially paraneurotransplants, which contain scattered and congenital neuroendocrine cells. The origin of tissue in thymic neuroendocrine tumours is unknown. Neuroendocrine cancer is often associated with thymic scale cancer and, in occasional cases, can be mixed with thymus tumours, which supports the idea that common thymic epithelial precursor cells were the ancestors of thymic neuroendocrine cancer (5).

### Pathological Type

The WHO has carried out a new classification of neuroendocrine tumours in the thymus and proposed diagnostic criteria, which are of great significance for clinicopathological diagnosis. According to the recent WHO classification criteria, thymic neuroendocrine tumours are divided into the following 2 categories: well-differentiated neuroendocrine carcinoma, containing typical classic carcinoid and atypical carcinoid, and poorly differentiated neuroendocrine carcinoma, containing small cell carcinoma (SCC) and large cell neuroendocrine carcinoma (LCNEC). Thymic SCC is an advanced tumour composed of small cells with unclear boundaries. Tumour cells are egg-shaped and shuttle-shaped, and nuclear division is common. Its cellular morphology is indicative of small cell lung cancer (SCLC). The variant type is complex SCC, which combines non-SCC components, such as squamous cell carcinoma and adenocarcinoma (6).

### Aetiology

Approximately 25% of patients with thymus cancer have a MEN-1-positive family history, whereas 8% have thymus cancer in MENen-1 patients. As cases of thymic neuroendocrine cancer are concentrated in a small number of MEN-1 families and can manifest with various types of mutations other than 11q13 (MEN-1) site heterocyclic deficiency (LOH), mutations in genes and MEN-1 abnormalities (which may affect chromosome 1p tumour suppressor genes) lead to the occurrence of thymus cancer (7).

### Clinical Characteristics

Thymic neuroendocrine cancer often occurs in the anterior mediastinum, and cases have been reported to occur in ectopic thymus tissue next to the thyroid gland. Most poorly differentiated thymic neuroendocrine cancers and approximately 50% of differentiated neuroendocrine cancers can have local symptoms such as chest pain, cough, dyspnoea, or upper cavity vein syndrome. It is very rare for patients to have cancer-like syndrome (<1%). Notably, 17–30% of adults and more than 50% of children with thymus cancer were accompanied by Cushing syndrome due to the tumour cell secretion of adrenocorticotropic hormone (ACTH). It is noted that 10% of all ectopic ACTH syndromes are caused by thymus cancer. In fact, thymic SCC rarely causes Cushing syndrome (8).

This 71-year-old male patient was asymptomatic, and the tumours were incidentally observed on routine chest CT without any positive signs by physical examination. Similar to the case presented in this study, over one-third of patients are asymptomatic, which reduces the early detection rate to a certain extent.

**FIGURE 4:**
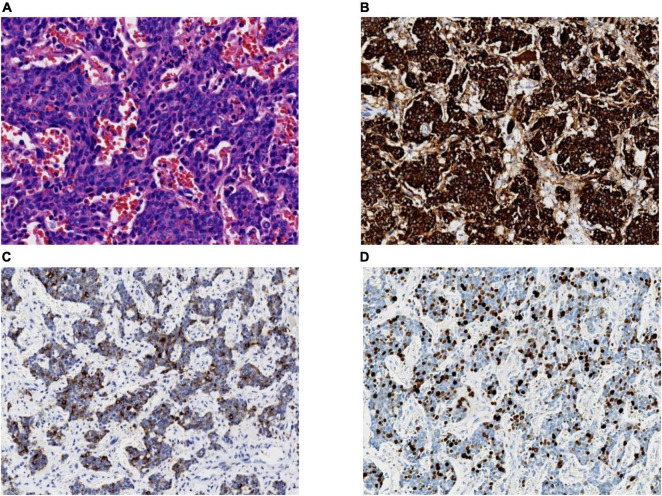
**(A)** A high-power view of the tumour biopsy under a light microscope. Hematoxylin and eosin staining (magnification, 40×); **(B)** synaptophysin staining (magnification, 20×); **(C)** chromogranin A staining (magnification, 20×); **(D)** Ki-67 staining. In total, ∼30% of the tumour cells are positive (magnification, 20×).

Treatments for thymic neuroendocrine cancer are very limited thus far. Surgery is the most effective treatment for thymic neuroendocrine cancer, and radical excision is the most critical factor for predicting the long-term survival of patients. Similar to the operation performed on this patient, radical excision often consists of en bloc resection of the tumour as well as the involved structures (9). The role of radiotherapy and chemotherapy in the treatment of thymic NEC is controversial. Radiotherapy and chemotherapy usually play secondary roles, while patients still have the possibility of surgical resection. Radiotherapy is recommended to prevent local recurrence of invasive tumours, and chemotherapy is usually used as an adjuvant treatment after surgery (10). Radiotherapy combined with chemotherapy in patients who do not qualify for surgery remains controversial because the effectiveness is difficult to evaluate. Fortunately, this patient was discovered in a timely manner and underwent surgical resection in time. Testing during the operation indicated that the tumour was highly malignant, and pathology results confirmed this after surgery. The tumour cells were small, with fewer than 3 quiescent lymphocytes, with few cytoplasmic cells that were densely crowded and arranged in nests and sheets. Abundant apoptotic debris and mitotic figures were found, and immunohistochemical stains were strongly positive for CK, Syn, CgA, and CD56 (Figure 4). Adenocarcinoma synaptophysin (Syn) and chromogranin (CgA) are currently recognised as neuroendocrine markers (7), and combined detection can improve the diagnosis rate. At the same time, electron microscope observation found that neuroendocrine granules (NSGs), tension fibrils, and desmosomes were of decisive significance in confirming neuroendocrine cancer. Depending on the four main types, typical carcinoid, atypical carcinoid, LCNEC, and SCC, and according to the immunohistochemical and morphological diagnostic criteria, the patient in this study was diagnosed with thymic SCNEC.

Morphologically, thymic SCNEC and small cell lung neuroendocrine carcinoma cannot be distinguished, and some immunological markers must be used to distinguish these two. TTF-1 may help to differentiate lung and thymic SCC. Most thymic carcinoids and neuroendocrine carcinomas do not express TTF-1, while 50–75% of lung neuroendocrine carcinomas express TTF-1. Carcinoids located in the mediastinum do not express TTF-1, which cannot be excluded from carcinoids metastasised from the gastrointestinal tract and pancreas because carcinoids of the gastrointestinal tract and pancreas often do not express TTF-1. The patient underwent a colour Doppler ultrasound examination of the abdomen as well as brain CT, and the inspection results showed no obvious abnormalities. In addition, the immunohistochemistry results showed TTF-1 (–) in this patient. According to related literature reports, Syn and CgA are not expressed in adenocarcinoma but are in thymic carcinoids and neuroendocrine carcinomas. Syn and CgA were all positively expressed according to the pathological results. These results all support that this tumour is of thymic origin, which is rare in the clinic. The patient was discharged smoothly, and the follow-up treatment was radiotherapy combined with chemotherapy, according to the advice from Shanghai Chest Hospital. We will continue to pay close attention to the patient’s condition.

In general, thymic SCNEC is a type of highly malignant tumour, is poorly differentiated, is easy to metastasise, and has a poor prognosis. The diagnosis relies on pathology and immunohistochemistry. The treatment is mainly combined with surgery, radiotherapy, and chemotherapy combined with biological therapy, but the effect is low. Therefore, early detection, diagnosis, and early treatment are the keys to improve prognosis. For malignant tumours that easily invade proximal structures, surgical planning under the guidance of three-dimensional reconstruction modelling before surgery will greatly reduce the risk of surgery and increase the complete resection rate, thereby improving the prognosis of patients. The three-dimensional reconstruction modelling technology is rarely used in clinical practice for mediastinal tumours and can provide surgeons with a safer and more intuitive operation plan.

## Data Availability Statement

The original contributions presented in this study are included in the article/Supplementary Material, further inquiries can be directed to the corresponding author.

## Author Contributions

TX, HL, and JG made contributions to the surgery. FL made contributions to the three-dimensional reconstruction which played an important role during the surgical planning. LC contributed prominently to the pathology discussion section of the article. All authors contributed to the article and approved the submitted version.

## Conflict of Interest

The authors declare that the research was conducted in the absence of any commercial or financial relationships that could be construed as a potential conflict of interest.

## Publisher’s Note

All claims expressed in this article are solely those of the authors and do not necessarily represent those of their affiliated organizations, or those of the publisher, the editors and the reviewers. Any product that may be evaluated in this article, or claim that may be made by its manufacturer, is not guaranteed or endorsed by the publisher.
